# Ethanol Dehydrogenase I Contributes to Growth and Sporulation Under Low Oxygen Condition via Detoxification of Acetaldehyde in *Metarhizium acridum*

**DOI:** 10.3389/fmicb.2018.01932

**Published:** 2018-08-21

**Authors:** Erhao Zhang, Yueqing Cao, Yuxian Xia

**Affiliations:** ^1^School of Life Sciences, Chongqing University, Chongqing, China; ^2^Chongqing Engineering Research Center for Fungal Insecticides, Chongqing, China; ^3^Key Laboratory of Gene Function and Regulation Technologies under Chongqing Municipal Education Commission, Chongqing, China

**Keywords:** ethanol dehydrogenase, conidiation, growth, entomopathogenic fungi, hypoxic condition

## Abstract

The entomopathogenic fungi encounter hypoxic conditions in both nature and artificial culture. Alcohol dehydrogenases (ADHs) are a group of oxidoreductases that occur in many organisms. Here we demonstrate that an alcohol dehydrogenase I, *MaADH1*, in the locust-specific fungal pathogen, *Metarhizium acridum*, functions in acetaldehyde detoxification mechanism under hypoxic conditions in growth and sporulation. The *MaADH1* was highly expressed in sporulation stage under hypoxic conditions. Compared with a wild-type strain, the Δ*MaADH1* mutant showed inhibited growth and sporulation under hypoxic conditions, but no impairment under normal conditions. Under hypoxic conditions, Δ*MaADH1* mutant produced significant decreased alcohol, but significant increased acetaldehyde compared to wild type. *M. acridum* was sensitive to exogenous acetaldehyde, exhibiting an inhibited growth and sporulation with acetaldehyde added in the medium. MaADH1 did not affect virulence. Our results indicated that the *MaADH1* was critical to growth and sporulation under hypoxic stress by detoxification of acetaldehyde in *M. acridum*.

## Introduction

Entomopathogenic fungi are one of the widespread organisms belonging to different systematic groups. About 1000 species of these fungi have been recorded as insect pathogens (Fang et al., 2012). Entomopathogenic fungi are distributed throughout the world, from the plateaus to the plains, and from the deserts to the swamps (Zimmermann, 2007). Hence entomopathogenic fungi are exposed to a variety of environmental stresses, including hypoxia, heat, UV radiation, etc. (Ortiz-Urquiza and Keyhani, 2015). However, altitude has no impact on the presence of entomopathogenic fungi even in a range up to 5200 m where oxygen was only 50% of the sea level value (Peacock, 1998; Quesada-Moraga et al., 2007; Sun and Liu, 2008). *Beauveria* and *Metarhizium* had been reported in Nepal and Tibet, where the oxygen availability was only 70% of the normal atmospheric levels value (Peacock, 1998; Yubak Dhoj et al., 2008; Freed et al., 2011), showing their ability to adapt to a wide range of oxygen levels. Thus, entomopathogenic fungi can grow and produce conidia under hypoxic conditions in nature, and they likely have mechanisms to sense and respond to hypoxic stress.

Entomopathogenic fungi are one of the most promising agents for biological control of pest insects. Some species have been developed to biopesticide products. Up to 2016 September, 51 commercial fungal biopesticide products from 58 countries around the world are available, which are mostly based on the aerial conidia for at least 12 species of insect fungi (de Faria and Wraight, 2007; Muñiz-Paredes et al., 2017). As a microbial pesticide, *M. acridum* was used for locust and grasshopper control in Africa, Australia and Asia (Hunter et al., 2001; Lomer et al., 2001; Peng et al., 2008). Aerial conidia of entomopathogenic fungi produced in solid culture are of lipophilic nature, a superb property for developing an oil-based formulation suitable for ultra-low volume (ULV) application in arid environments (Bateman et al., 1993; Burges, 1998; Lomer et al., 2001). As aerobic organisms of entomopathogenic fungi, aeration is an important factor for aerial conidia production (Muñiz-Paredes et al., 2017). However, the solid-state fermentation has some disadvantages, such as oxygen limitation due to poor diffusion of oxygen in solid substrate especially for mass production (Bartlett and Jaronski, 1998; Mienda et al., 2011). Therefore, aeration has a strong impact on the fungal growth and conidiation (Rahardjo et al., 2005). The increase of dissolved oxygen (DO) during sporulation in the liquid culture results in an increased blastospore production compared to normal condition (Issaly et al., 2005). In solid culture, conidia production was increased significantly under higher oxygen (26% O_2_) compared to normal oxygen condition (21% O_2_) (Tlecuitl-Beristain et al., 2010). Normally, entomopathogenic fungi encounter the challenges of hypoxic stress in nature or in artificial culture. However, up to now, the regulation mechanisms of hypoxic stress tolerance have not been explored in entomopathogenic fungi.

Alcohol dehydrogenases (ADHs) are a group of oxidoreductases that occur in many organisms facilitating the conversion between alcohols and aldehydes with the reduction of NAD+. Alcohol dehydrogenases are widespread in the animals, plants, fungi and bacteria. However, the ADH type and number varied in different species. For example, there are five ADH members (ADH1, ADH2, ADH3, ADH4, and ADH5), three in *Saccharomyces cerevisiae* (ADH1, ADH2, and ADH3) (de Smidt et al., 2008), two in *Aspergillus nidulans* (ADH1 and ADH3) (Reid and Fewson, 1994), and one in *Mucor circinelloides* (ADH1) (Rangel-Porras et al., 2005).

The functions of ADH members vary within the ADH family. In *Thermoanaerobacter ethanolicus*, three ADHs play different roles during ethanol formation (Zhou et al., 2017). In yeast, ADH1 is responsible for ethanol production from acetaldehyde and NADH, contributing to fungal growth in the presence of alcohol (Plapp et al., 2013). Yeast ADH2 converts the ethanol accumulating to acetaldehyde under anaerobic conditions (de Smidt et al., 2008). In plant pathogenic fungus *Fusarium oxysporum*, ADH1 disruption impaired growth under hypoxic conditions, diminished production of ethanol and affected the fungal disease development in tomato plants (Corrales Escobosa et al., 2011). In *A. fumigatus*, ADH could be induced by hypoxic condition, and influence fungal growth and pathogenesis (Grahl et al., 2011).

The *ADH* genes widely exist in the insect pathogenic fungi of *Metarhizium, Beauveria*, and *Cordyceps*. Few past studies were reported on the function of alcohol dehydrogenase in entomopathogenic fungi. In *M. anisopliae, ADH1* is expressed during infection process and required for virulence (Callejas-Negrete et al., 2015). In this study, we explored the function of the *MaADH1* gene by targeted disruption in *M. acridum*. Our results showed that Δ*MaADH1* had inhibited growth and sporulation under hypoxic conditions, while it had no impairment under normal oxygen conditions. When oxygen level was low, Δ*MaADH1* mutant produced decreased alcohol and increased acetaldehyde compared to wild type. Exogenous acetaldehyde had similar inhibition on sporulation as deletion of *MaADH1* in *M. acridum.* These data suggested that *MaADH1* was involved in hypoxic stress tolerance and contributed to conidiation under low oxygen condition.

## Materials and Methods

### Organisms and Culture Conditions

*Metarhizium acridum* CQMa102 is wild type strain that was obtained from China General Microbiological Culture Collection Center (CGMCC, No. 0877). The fungal strains were propagated in 1/4 SDAY medium, consisting of 1% dextrose, 0.25% mycological peptone, 0.5% yeast extract, and 2% agar (w/v). *Escherichia coli* DH5α (DingGuo, Beijing, China) was used for routine cloning.

### Semi-Quantitative Reverse Transcription (qRT)-PCR and qRT-PCR

Total RNAs were extracted from WT mycelia grown for 14 and 20 h days on plate, conidia (growing for 3 days), germinated conidia, appressoria formed on locust wing, and hyphal bodies grown for 5 days in hemolymph of infected locusts (Luo et al., 2013). Appressorium induction and hyphal body collection were performed as described previously (He and Xia, 2009). Total RNAs were extracted using TRIzol reagent (Invitrogen, United States). For first strand cDNA synthesis, 1 μg total RNA was applied with an oligo-dT primer using the PrimeScript^TM^ RT Master Mix (TaKaRa, Dalian, China). Semi-quantitative RT-PCR and qRT-PCR were performed using primers ADH1-EF and ADH1-ER. cDNA synthesis and qRT-PCR were performed using the method described by Luo et al. (2013). All PCR amplifications were conducted in triplicate, the glyceraldehyde-3-phosphate dehydrogenase gene (gpd) was used as an internal control. Transcript ratios of target gene were evaluated using the 2^-ΔΔCT^ method (Livak and Schmittgen, 2001). Primer sequences are listed in **Supplementary Table S1**.

### *MaADH1* Deletion, Complementation, and Over-Expression

The *MaADH1* gene was disrupted by homologous recombination. About 1 kb upstream and 1 kb downstream sequences of *MaADH1* open reading frame (ORF) were amplified with primer pairs LF/LR and RF/RR from *M. acridum* genomic DNA. Amplification fragments were inserted into pK2-pB (Luo et al., 2013) separated by a selection marker Bar cassette. The recombinant plasmid was transformed into *M. acridum* with *Agrobacterium tumefaciens* according to Fang et al. (2004). Transformants were screened by PCR with primers L1/PR and BF/R1 and confirmed by Southern blot. A plasmid for complementation was generated using primers HF/HR to amplify the entire *MaADH1* gene including 2 kb of the *MaADH1* promoter, the coding sequence, and the terminator. The amplified fragment was inserted into pK2-EGFP-Sur containing the chorimuron ethyl resistance gene Sur (Luo et al., 2013). The pK2-Sur-ADH1-EGFP construct was transformed into the Δ*MaADH1*. Transformants were screened on Czapek-dox plates supplemented with 20 μg/ml chorimuron ethyl (Sigma, Bellefonte, PA, United States) and confirmed by DNA blots. Primers used for transformants construction are listed in **Supplementary Table S1**.

### Growth Under Different Oxygen Condition

To examine the role of MaADH1 on fungal growth under different DO condition, WT, *ΔMaADH1*, and complemented strain (CP) were grown in liquid culture under normal dissolved-oxygen and low DO condition, respectively. Conidia were inoculated into 1/4 SDY liquid medium with a concentration of 1 × 10^5^ conidia/ml in 50 ml centrifuge tubes. Centrifuge tubes were sealed with ventilated films for normal oxygen condition. To get lower DO, 20 μl liquid CO_2_ was added in the culture mixture to obtain about 18% oxygen concentration at the initial time of growth (80% of normal oxygen level), then the tubes were sealed with caps and shaken at 250 rpm at 28°C. The fungal cultures were collected by centrifugation every 12 h during a 5-day growing period. The pellets were weighed and compared among fungal strains. The transcription of *MaADH1* and ADHs enzyme activity were also determined in these samples. Total RNAs were extracted from these fungal cultures for transcription level analysis of *MaADH1* by qRT-PCR as described above. The DO in liquid culture was detected with DO electrode (Mettler Toledo, Inpro6000, Switzerland).

### ADH Activity Assay

The ADH activity assays were performed in reaction mixtures containing 25 μl cell-free fractions, 1.0 ml semicarbazide buffer (pH 8.7), 1.5 mM NAD+ solutions and 10 mM ethanol. The reduction of NAD+ was monitored by the increased absorbance at 340 nm. One unit of enzyme activity was defined as the amount of enzyme catalyzing the reduction of 1 μmol NAD+ per min at 25°C. Specific activity was expressed as units (U) per mg of protein. Protein concentration was determined using the BCA protein assay kit (Beyotime, China)

### Sporulation Determination

Sporulation under hypoxic condition was determined in liquid medium and on solid rice grain. Fungal strains were grown in liquid medium under hypoxic condition as described above. After growing for 3 days, the fungal culture was collected and filtered with four layers of lens tissue to remove mycelia. Blastospore concentration in filtered solution was determined by hemocytometer under microscope. Two-stage technique was applied in solid-state fermentation, which involves submerged liquid fermentation and solid substrate fermentation. Rice grains were soaked in water for 5 h, and then were autoclaved after removing water. Three-day fungal culture grown in liquid 1/4 SDY medium (1 × 10^6^ spore/ml) under normal condition was mixed thoroughly with grains to a final concentration of 10% (V/W). The mixture was then filled into sealed glass tubes (diameter: 3.5 cm, height: 11 cm), and then fermented at 28°C for 14 days to produce conidia. The rice grains were shaken once every 2 days to prevent the grains sticking together. Fermented rice (0.25 g) was soaked in 1 ml H_2_O, and vortexed to wash off the conidia from rice grains. Conidia number was determined by hemocytometer under microscope.

### Alcohol and Acetaldehyde Determination

Alcohol and acetaldehyde determination was performed as described previously with minor modifications (Bernt and Gutmann, 1974; Rotariu et al., 2004). Fungal growth in liquid 1/4 SDY under hypoxic condition were as described above. The cultures were collected every 12 h during a 5-day growing period and then centrifuged to collect the supernatant. For alcohol determination, supernatant of 25 μl was mixed thoroughly with 1.0 ml semicarbazide buffer (pH 8.7) and 25 μl NAD+ solutions (Solarbio, Beijing, China). Alcohol dehydrogenase solution (5 μl) sourced from *S. cerevisiae* (Solarbio, Beijing, China) was added to the mixture and then incubated at 25°C for 30 min. The absorbance of the reaction mixture was read at 340 nm. Standard curve was generated using 0–0.04% (v/v) ethanol solutions.

For acetaldehyde determination, 25 μl of sample solution was added to a tube containing 300 μl Tris–HCl buffer (1 M, pH 8.0) and 25 μl NAD+ solutions (1 mM). Acetaldehyde dehydrogenase (E.C 1.2.1.5) (5 μl) was added to the mixture and then incubated at 25°C for 30 min. The absorbance of the reaction mixture was read at 340 nm. Standard curve was generated using 0–0.04% (v/v) acetaldehyde solutions prepared with phosphate buffer pH 9.0 containing 0.1 M KCl.

### Effect of Acetaldehyde on Fungal Growth

To investigate the effects of acetaldehyde on fungal growth, wild type strain was grown in liquid or solid medium containing 0.01 or 0.1% acetaldehyde. For liquid culture, conidia were inoculated in 1/4 SDY in a 50 ml centrifuge tube at a final concentration of 1 × 10^5^ conidia/ml, and then shaken at 28°C with 250 rpm for 4 days. Blastospore production was determined daily. For culture on plate, conidia (1 × 10^6^ conidia/ml) were spotted or spread on 1/4 SDAY plate and grown for 14 days. Conidia production was determined every 3 days as described previously (Liu et al., 2010). The phenotype of fungal colonies was shown on 6th day of growth.

### Germination, Stress Tolerance, and Bioassay

Germination rate was determined on normal medium (1/4 SDAY) and host insect wing [*Locusta migratoria manilensis* (Meyen)] as described previously (Liu et al., 2010).

Tolerance to heat and ultraviolet radiation was determined according to Liu et al. (2010). IT_50_ (time for 50% inhibition in germination rate by heat or UV irradiation) was compared among WT, Δ*MaADH1*, and the CP.

Bioassay was performed using fifth instar nymphs of *L. migratoria* as described previously (Cao et al., 2012). Conidia suspensions (5 μl) were topically inoculated on pronotum (1 × 10^7^ conidia/ml liquid paraffin oil). Half-lethal time (LT_50_) was calculated and compared between WT and Δ*MaADH1*.

### Data Analysis

Conidiation production, enzyme activity, ethanol, and acetaldehyde concentration, germination rate, IT_50_ and LT_50_, were statistically analyzed using one-way analysis of variance model (SPSS 16.0; SPSS Inc., Chicago, IL, United States). Tukey’s honestly significant difference test was used to separate means at *p* = 0.05.

## Results

### Gene Cloning and Molecular Characterization

The full length of cDNA of *MaADH1* is 1062 bp (Accession number KX021843). It encodes a predicted protein of 353 amino acids residues, with a deduced molecular weight of 37 KDa and a pI of 7.61. The SignalP 3.0 program revealed no signal sequence, suggesting that MaADH1 is a cell-bound protein. MaADH1 contains typical ethanol dehydrogenase structural domains including NAD+ binding domain, Zn^2+^ binding site and ADH1 activity domain (**Supplementary Figure S1A**). Phylogenic analysis indicated that MaADH1 protein was closely related to ADH1 from the entomopathogenic fungi *M. anisopliae* (identity 94%) and *Beauveria bassiana* (identity 73%) (**Supplementary Figure S1B**).

### *MaADH1* Is Induced in Hypoxic Condition

**FIGURE 1:**
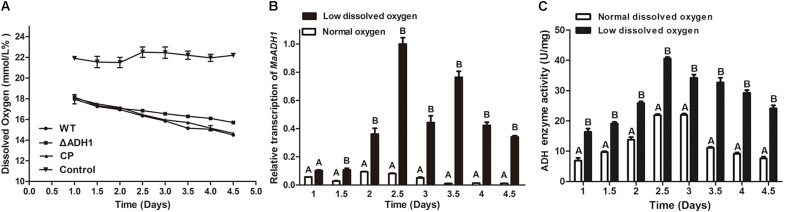
Transcription and enzyme activity. **(A)** Dissolved oxygen under normal and low oxygen condition. **(B)** Relative expression of *MaADH1* under hypoxic condition and normal condition. **(C)** ADH activity at different growth time under hypoxic condition and normal condition. A, B indicate significant difference at *p* < 0.01.

The concentration of DO in culture fluctuated around the value of 21% under normal condition during growth period (**Figure 1A**). Under low oxygen condition, DO in culture was about 18% at the initial time and then gradually decreased along with the fungal growth. However, DO in Δ*MaADH1* culture was slightly higher than WT and CP strains (**Figure 1A**). In 1/4 SDY liquid medium, the *MaADH1* had significantly increased transcription under low DO condition, with up to 10 times higher as compared to normal oxygen condition (**Figure 1B**). Transcription of *MaADH1* gene was drastically increased after 2 days of growth under hypoxic condition, while normal oxygen condition had slight effect on *MaADH1* transcription in this period (**Figure 1B**). Blastospores were produced substantially in liquid media during this period. These results demonstrated that *MaADH1* might have a function in sporulation, especially sporulation under hypoxic condition.

Alcohol dehydrogenases enzyme activities were also determined in *M. acridum* under low and normal DO conditions. ADH activity showed a similar trend as the transcription results of *MaADH1* (**Figure 1C**). The specific activity of ADHs was significantly increased under low DO condition compared to aerobic condition. The ADH enzyme activity increased from 1st day and reached to peak value of 40.5 ± 1.5 U/mg after growing for 2.5 days under hypoxic condition, almost 2 times higher than that under aerobic condition (21.5 ± 0.9 U/mg). A drastic decrease in ADHs activity was found at 3.5 days under aerobic condition, showing about 50% decrease compared to the peak value, while a 20% decrease was found at 3.5 day under hypoxic condition (**Figure 1C**). However, compared to transcription results of *MaADH1*, ADH enzyme activity did not have such drastic difference between normal and hypoxic condition (10 times versus 2 times increase) (**Figure 1C**). We supposed that this was due to the total ADH activity we determined in this study, and some other ADH members might require different biochemical conditions to measure the activity or have relative higher activity under normal oxygen condition, thus obscuring the difference in total ADH activity under normal and hypoxic condition.

### *MaADH1* Is Highly Expressed in Sporulation

**FIGURE 2:**
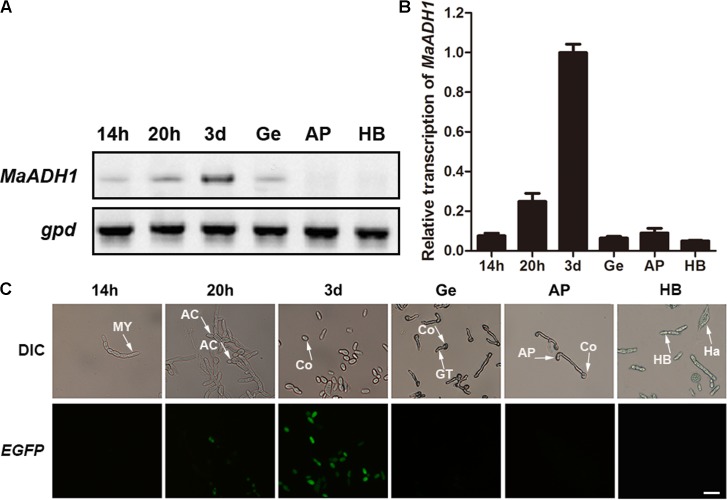
Expression pattern of *MaADH1* by semi-quantitative RT-PCR **(A)** and qRT-PCR **(B)** analysis of transcription of the *MaADH1* gene on different life stages. **(C)** Fluorescence of *MaADH1-EGFP* in different stage. Ge, germination; AP, appressorium; HB, hyphal body; MY, Mycelium; AC, Arthroconidia; Co, conidia; GT, germ tube; Ha, hemocyte. Scale bar = 10 μm.

The expression of *MaADH1* at different stages of the life cycle and under low DO condition in liquid culture was analyzed by semi-quantitative RT-PCR and qRT-PCR. On 1/4 SDAY plate, the *MaADH1* was highly transcribed in sporulation stage (3-day culture on plate) and poorly transcribed in mycelium, appressorium and hyphal body formation stages (**Figures 2A,B**). Consistent with the transcription results, MaADH1-EGFP fusion expression showed that the fungus had strong green fluorescence in sporulation stage on the 3rd day grown on plate, while weak or no signal was found in mycelia, germ tubes, appressorium and hyphal body (**Figure 2C**).

### *MaADH1* Contributes Fungal Growth and Conidiation Under Low Oxygen Condition

**FIGURE 3:**
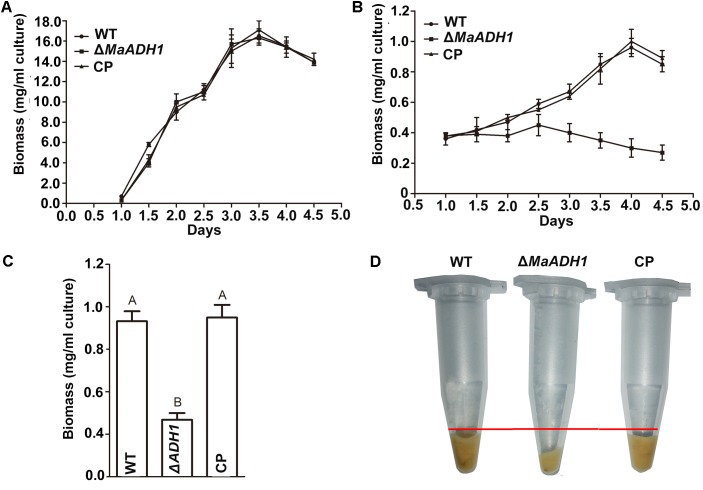
Biomass analysis. **(A)** Time course study of dry weight under normal oxygen condition. **(B)** Biomass under low dissolved-oxygen condition. Dry weight **(C)** and pellets **(D)** on 3rd day under low dissolved-oxygen condition. Error bars are standard deviations of three trials. A, B indicate significant difference at *p* < 0.01.

Targeted gene disruption of *MaADH1* was generated by homologous recombination (**Supplementary Figure S2A**). Correct integration events in transformants of the deletion and subsequent complementation mutants were confirmed by Southern blotting (**Supplementary Figure S2B**). To examine the role of *MaADH1* in fungal growth, biomass of fungal strains in liquid culture was analyzed under normal dissolved-oxygen and low DO condition, respectively. Results showed that biomass of Δ*MaADH1* had no significant difference compared with WT and CP under normal oxygen condition (**Figure 3A**), while decreased significantly after growing for 3 days under low oxygen condition (**Figures 3B,C**). The pellet of Δ*MaADH1* fungal culture was obviously less than WT and CP strains (**Figure 3D**). These results indicated that *MaADH1* affected growth of *M. acridum* in liquid medium under low DO condition.

**FIGURE 4:**
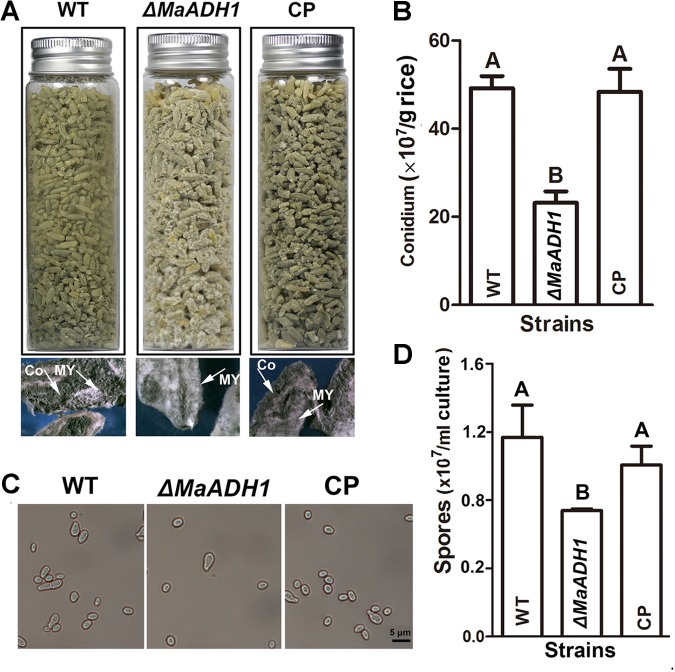
Sporulation under hypoxic conditions. **(A)** Conidiation after growing on rice under hypoxic condition for 12 days. CO: conidium; MY: mycelia. **(B)** Quantification of conidiation on rice. **(C)** Sporulation in liquid medium under hypoxic condition **(D)** Quantitative sporulation in liquid medium under hypoxic condition. All experiments were repeated at least three times. Error bars, SD. A, B indicate significant difference at *p* < 0.01.

Conidiation of fungal strains on rice grains under low oxygen condition showed that rice grains in both WT and CP strains groups were covered with dark green conidia, while grains of Δ*MaADH1* group were covered with light-green conidia (**Figure 4A**). Microscopic analysis revealed that more white mycelia were found on rice surface in Δ*MaADH1* group compared to WT (**Figure 4A**). Quantitative conidiation analysis showed that Δ*MaADH1* had significantly decreased conidia production, almost 50% less than wild type strain, suggesting that *MaADH1* also contributed to aerial conidiation in *M. acridum* under hypoxic condition (**Figure 4B**). Sporulation in liquid culture exhibited the same decreased trend as on rice grains in Δ*MaADH1* under hypoxic condition (**Figures 4C,D**).

MaADH1 did not affect conidia germination, heat and UV stress tolerance, and virulence (data not shown).

### *MaADH1* Regulates Acetaldehyde Metabolism in *M. acridum*

**FIGURE 5:**
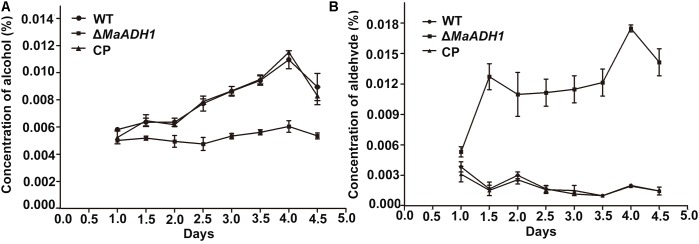
Alcohol and acetaldehyde production under low oxygen condition. Concentration of alcohol **(A)** and acetaldehyde **(B)** in liquid medium at the different cultural time. Error bars are standard deviations of three trials.

Alcohol dehydrogenases constitute a large family of enzymes responsible for the reversible oxidation of alcohols to aldehydes with the concomitant reduction of NAD+ or NADP+. To further explore the roles of *MaADH1* under low oxygen condition, the ethanol and acetaldehyde in the liquid culture were measured. Compared to wild type, the Δ*MaADH1* culture had significant decreased alcohol concentration (about 0.005% in Δ*MaADH1* versus to 0.008% in WT) (**Figure 5A**) and significant increased concentration of acetaldehyde (about 0.01% in Δ*MaADH1* versus to 0.002% in WT) (**Figure 5B**). Considering the inhibited growth results of Δ*MaADH1*, it could be speculated that acetaldehyde accumulation in the culture was toxic to *M. acridum*.

### Acetaldehyde Affected Conidiation in *M. acridum*

**FIGURE 6:**
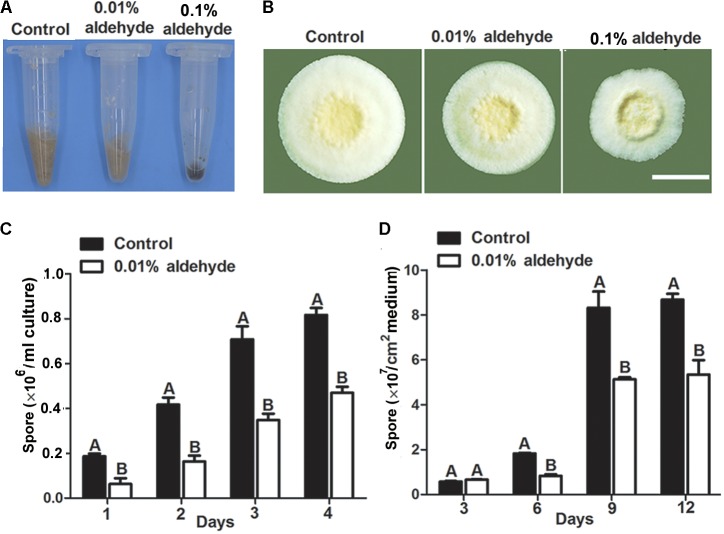
Effect of aldehyde on conidiation under normal condition. **(A)** Sporulation in the 1/4 SDA liquid culture medium containing aldehyde **(B)** Conidiation on 1/4 SDAY solid medium with aldehyde. **(C)** Sporulation quantification in liquid medium with 0.01% aldehyde **(D)** Sporulation quantification on solid medium with 0.01% aldehyde. All experiments were repeated at least three times. Error bars, SD. A, B indicate significant difference at *p* < 0.01.

To further analysis the influence of acetaldehyde on growth and sporulation, wild type strain was inoculated on 1/4 SDAY plate and in liquid media containing acetaldehyde (0.01 or 0.1%, v/v). Results showed that acetaldehyde inhibited the growth of *M. acridum* at concentrations of both 0.1 and 0.01% on plate (**Figure 6A**) or in liquid medium (**Figure 6B**), suggesting the acetaldehyde accumulation caused by *MaADH1* deletion was sufficient to inhibit fungal growth. Compared to control, sporulation production was decreased significantly both in liquid (**Figure 6C**) and on solid medium (**Figure 6D**) containing acetaldehyde, showing about 50% decrease during the inspection period. These results suggested that acetaldehyde had negative effect on sporulation in *M. acridum*.

## Disscussion

In this study, we identified an *ADH1* gene from the entomopathogenic fungus *M. acridum*, also explored its function in sporulation and growth under low or normal oxygen condition. Our results showed that the *MaADH1* gene contributed to growth and sporulation under low oxygen condition via detoxification of acetaldehyde. However, this gene was not involved in virulence and UV/heat stress tolerance in *M. acridum*.

MaADH1 mainly accounted for conversion from acetaldehyde to ethanol under hypoxic condition in *M. acridum*. Similar as reported in some other fungi (Leskovac et al., 2002; Rangel-Porras et al., 2005), The *MaADH1* had an up-regulated transcription and increased enzyme activity under low oxygen condition, suggesting its role under micro-oxygen conditions. Our data revealed a decrease in ethanol production and an increase in acetaldehyde production in Δ*MaADH1* culture, indicating a block in ethanol production from acetaldehyde under hypoxic condition when the *MaADH1* gene was impaired. This physiological function was consistent with earlier reports in some fungi, such as *S. cerevisiae* (Leskovac et al., 2002; de Smidt et al., 2008), *Candida maltose* (Lin et al., 2010), *F. oxysporum* (Corrales Escobosa et al., 2011), *M. anisopliae* (Callejas-Negrete et al., 2015), and *M. circinelloides* (Rangel-Porras et al., 2005).

ADH1 is an enzyme strategy to detoxify acetaldehyde under low oxygen condition in *M. acridum*. Acetaldehyde is an inhibitor of a wide range of metabolic activities and is toxic to fungi (Aranda and del Olmo, 2004). Acetaldehyde at high concentrations can even stop yeast cell growth (Stanley et al., 1993). Consistence to these reports, our results showed that acetaldehyde had strong adverse effect on growth in *M. acridum* (**Figure 5**). ADH1 is a fermentative enzyme to catalyze the transformation of acetaldehyde to ethanol, reducing the accumulation of acetaldehyde in cells. In our study, deletion of the *MaADH1* gene resulted in accumulation of acetaldehyde (reach to a concentration of 0.01%) in culture (**Figure 5**) and inhibited growth and sporulation. The inhibition effect was in accordance with that of exogenous acetaldehyde (at a concentration of 0.01%) on the growth of wild type strain (**Figure 6**). It is therefore reasonably supposed that MaADH1 played a major role in overcoming acetaldehyde toxicity in the liquid or solid-state fermentation in *M. acridum*.

The ability to sense and respond to changes in oxygen is essential for the survival of prokaryotic and eukaryotic organisms (Giaccia et al., 2004). Our data showed that fungal blastospore production in liquid culture appeared to be clearly related to the DO concentration. Sporulation was significantly decreased with low DO concentration (**Figure 3**). These results were in accordance with the previous report in *M. flavoviride* (Issaly et al., 2005). Ethanol could be detected when oxygen was depleted in part of the solid medium (Oostra et al., 2001), which also agreed to our data (**Figure 5**). Therefore, microbes would develop a strategy to decrease the adverse effect of hypoxic condition. As a group of fermentative enzymes, ADHs might play a role in this process. The physiological functions vary among different ADH members. In *Thermoanaerobacter ethanolicus*, AdhE primarily functions in acetaldehyde production, and AdhB has high activity for ethanol production, but does not contribute to acetaldehyde production (Zhou et al., 2017). In yeast, ADH1 and ADH2 are responsible for the inverse conversion between ethanol and acetaldehyde (de Smidt et al., 2008). Therefore, ADHs members might coordinate to overcome the hypoxic stress. Our data revealed that *MaADH1* was induced in conidiation process under low oxygen condition, showing a much higher transcription compared to normal oxygen condition (**Figure 2**). This has also been reported in other ADH members in *A. fumigatus* (Grahl et al., 2011). Oxygen limitation has been the major concern in fungal solid-state fermentation (Bartlett and Jaronski, 1998; Mienda et al., 2011). Disruption of *MaADH1* resulted in decreased conidiation on rice grain (**Figure 4**), suggesting a role of *MaADH1* under hypoxic condition. Better understanding the regulation mechanisms and comprehensive utilization of ADH members in engineered strain construction could improve the fungal fermentation, especially in high density fermentation.

*ADH1* did not contribute to virulence in *M. acridum*. The infection process of entomopathogenic fungi involves attachment of conidia to the insect cuticle, germination of conidia, development of appressorium, formation of the penetration peg to penetrate the insects’ cuticle and killing the host insect by the growth of invasive hyphae in hemolymph and toxin produced by the fungi (Clarkson and Charnley, 1996). Our results revealed that ADH1 had very low transcription during germination and almost no transcription in appressoria and hyphal bodies. Accordingly, no fluorescence was detected in hyphal bodies and appressoria for ADH1-EGFP fused expression in *M. acridum*, suggesting ADH1 did not involve in pathogenicity process. Bioassay results against locusts were in accordance with the transcription profiles of *MaADH1*, showing no contribution of ADH1 to virulence (data now shown). However, our results were not consistent with the findings in *M. anisopliae*, which ADH1 is expressed during insect colonization and required for full virulence (Callejas-Negrete et al., 2015). ADH1 is also involved in virulence in plant pathogenic fungus *F. oxysporum* (Corrales Escobosa et al., 2011) and human pathogenic fungus *C. albicans* (Ku et al., 2017) and *A. fumigatus* (Grahl et al., 2011). This variation in ADH1 function illustrates that ADH1 might have distinct roles for a pathogenic fungi during interactions with host organisms.

In summary, our results demonstrated that the function of MaADH1 was to convert acetaldehyde to alcohol and eliminate side effect of acetaldehyde on fungal growth and sporulation under hypoxic conditions. Further better understanding the function and regulation of the *MaADH1* may potentially help to improve high density fermentation in entomopathogenic fungi.

## Author Contributions

EZ conducted the main experiment and wrote the manuscript. YX and YC conceived and designed the experiments. EZ and YC analyzed the data. YX and YC provided technical oversight and critical manuscript review and editing.

## Conflict of Interest Statement

The authors declare that the research was conducted in the absence of any commercial or financial relationships that could be construed as a potential conflict of interest.
