# Supplementary material for: Ethanol Dehydrogenase I Contributes to Growth and Sporulation Under Low Oxygen Condition via Detoxification of Acetaldehyde in Metarhizium acridum
Source: Front Microbiol. 2018 Aug 21;9:1932. doi: 10.3389/fmicb.2018.01932 (PMC6110892; doi:10.3389/fmicb.2018.01932)
Supplement: Supplementary file 1 [file Presentation_1.PDF]

Ethanol dehydrogenase I contributes to growth and sporulation under low oxygen condition via detoxification of acetaldehyde in *Metarhizium acridum*

Erhao Zhang 1,2,3, Yueqing Cao 1,2,3 *, Yuxian Xia1,2,3 *

1 School of Life Sciences, Chongqing University, Chongqing, China

2 Chongqing Engineering Research Center for Fungal Insecticides, Chongqing, China

3 Key Laboratory of Gene Function and Regulation Technologies under Chongqing Municipal Education Commission, Chongqing, China

Correspondence:

Yuxian Xia:

Email: yuxianxia@cqu.edu.cn

Yueqing Cao:

Email: yueqingcao@cqu.edu.cn

Table S1 Primers used in this study

Primer name	Sequence 5' to 3'
PADH1-ALL-F	gacggccagtgccaagctTTTCGACTGGGCTGCGAG
PADH1-ALL-R	ccttgctcaccatggatccTTTGTCAAGTGTTTCGTGGC
PADH1-Del 1-F	acgacggccagtgccaagctTTAGCGTGCGGTTGCGTG
PADH1-Del2-F	gacggccagtgccaagctATTTCCTTTTCCCCGCAGAC
PADH1-Del3-F	gacggccagtgccaagctTGCAGGTGTCGAAAGCTACGA
PADH1-Del4-F	gacggccagtgccaagctCAACGGAGCAAATCTTACC
PADH1-Del5-F	gacggccagtgccaagctACGTACTTAAGTATGTTTTGC
PADH1-Del6-F	gacggccagtgccaagctATGATACTCTCGTAGATCGTTTCCA
PADH1-Del7-F	gacggccagtgccaagctCGTCTTGTATATAAATGGCTG
LB-ADH1-F	gacggccagtgccaagctACGCCACTGTTATTTGCTG
LB-ADH1-R	cggatccctcgagtctagCTTGCTTTGGACGATTGC
RB-ADH1-F	gctggccgcccatgggatAGACCGCCTCAACAATAA
RB-ADH1-R	atgacatgattacgaattGACTCCTCCCTCAAGACC
CP-ADH1-F	gacggccagtgccaagctTGAGAGTAACTGGGTATTA
CP-ADH1-R	ccttgctcaccatggatccTTTGCTGGTGTCAACAAC
YZ-ADH1-F	TGCTGGCAAGAGTAGTCGG
YZ-ADH1-R	CTTGCCCTTGCTGCTTCTT
RT-gpd-F	GACTGCCCGCATTGAGAAG
RT-gpd-R	AGATGGAGGAGTGGGTGTTG
RT-EGFP-F	CACCATCTTCTTCAAGGA
RT-EGFP-R	GTGGCTGTTGTAGTTGTA
RT-ADH1-F	ACTGGAGGAGCCGTCGTATA
RT-ADH1-R	GGAACCGTTCAGCCACTT
ADH1-S-F	TGCCAATGCCCGTCCTGC
ADH1-S-R	CTTGCTTTGGACGATTGC
ADH1-F	ATGACCGCCAACAAGATC
ADH1-R	TTTGCTGGTGTCAACAAC
EGFP-VR	CGATGCGGTTCACCAGGGTGT

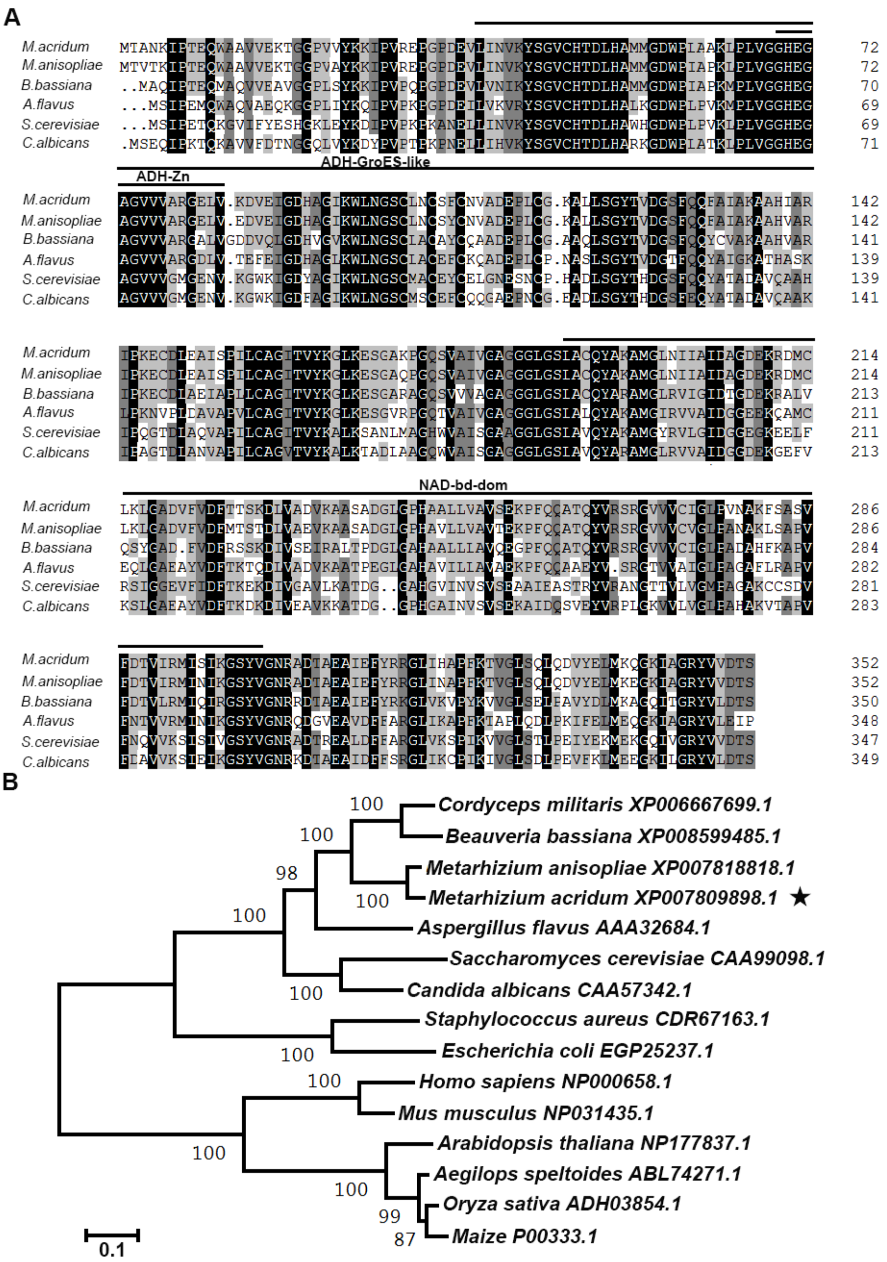

Figure S1 Sequence analysis of the *MaADH1* gene. (A) Conserved domain of *ADH1* in different species. *MaADH1* have three typical domains, ADH-Zn-CS (Zn binding site), ADH-GroEs-Like (substrate binding site) and NAD-bd-dom (NAD binding site) (B) Phylogenetic analysis of ADH1 homologous with MEGA 4.0 (Tamura et al. 2007).

Tamura K, Dudley J, Nei M, Kumar S. (2007) Mega4: Molecular Evolutionary Genetics Analysis (MEGA) software version 4.0. Mol Biol Evol 24:1596-1599

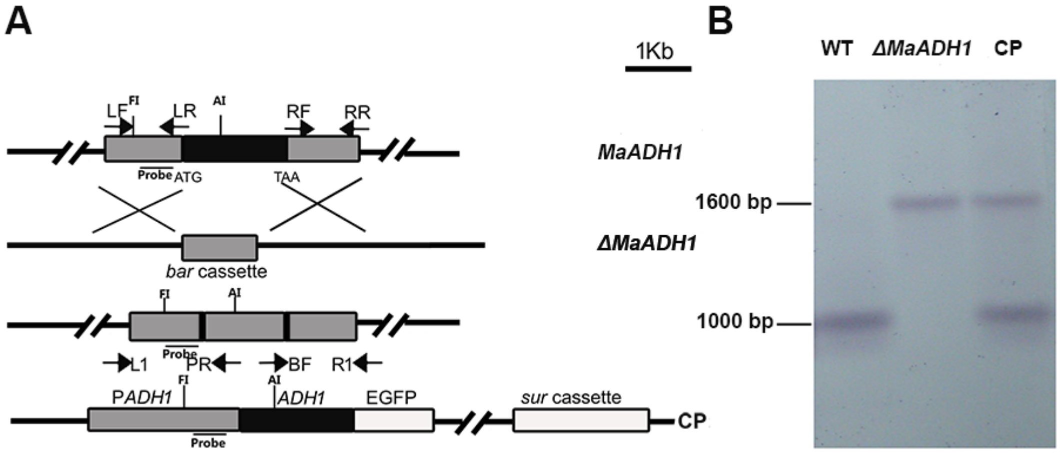

Figure S2 Construction and identification of Δ*MaADH1* and complemented strains.(A) Restriction maps of the MaADH1 locus, replacement vector pK2-PB-ADH1, and complementary cassette. FI: *Fba*I; AI: *Aor*51HI; *PADH1*: *MaADH1* promoter. LF/LR, RF/RR, L1/PR, R1/BF are primer pairs used in engineering strains construction and identification. (B) Southern blot. Genomic DNA from WT, irs use and complemented strains were digested with *Fba*I and *Aor*51HI. Probe, a 409 bp fragment of the *MaADH1* upstream flanking sequence was amplified with TF/TR listed in Table S1
